# Internet-Based Cognitive Behavior Therapy for Procrastination: Study Protocol for a Randomized Controlled Trial

**DOI:** 10.2196/resprot.2801

**Published:** 2013-11-12

**Authors:** Alexander Rozental, Per Carlbring

**Affiliations:** ^1^Division of Clinical PsychologyDepartment of PsychologyUniversity of StockholmStockholmSweden

**Keywords:** procrastination, cognitive behavior therapy, Internet-administered, randomized controlled trial

## Abstract

**Background:**

Procrastination, to voluntarily delay an intended course of action despite expecting to be worse-off for the delay, is a persistent behavior pattern that can cause major psychological suffering. Approximately half of the student population and 15%-20% of the adult population are presumed having substantial difficulties due to chronic and recurrent procrastination in their everyday life. However, preconceptions and a lack of knowledge restrict the availability of adequate care. Cognitive behavior therapy (CBT) is often considered treatment of choice, although no clinical trials have previously been carried out.

**Objective:**

The aim of this study will be to test the effects of CBT for procrastination, and to investigate whether it can be delivered via the Internet.

**Methods:**

Participants will be recruited through advertisements in newspapers, other media, and the Internet. Only people residing in Sweden with access to the Internet and suffering from procrastination will be included in the study. A randomized controlled trial with a sample size of 150 participants divided into three groups will be utilized. The treatment group will consist of 50 participants receiving a 10-week CBT intervention with weekly therapist contact. A second treatment group with 50 participants receiving the same treatment, but without therapist contact, will also be employed. The intervention being used for the current study is derived from a self-help book for procrastination written by one of the authors (AR). It includes several CBT techniques commonly used for the treatment of procrastination (eg, behavioral activation, behavioral experiments, stimulus control, and psychoeducation on motivation and different work methods). A control group consisting of 50 participants on a wait-list control will be used to evaluate the effects of the CBT intervention. For ethical reasons, the participants in the control group will gain access to the same intervention following the 10-week treatment period, albeit without therapist contact.

**Results:**

The current study is believed to result in three important findings. First, a CBT intervention is assumed to be beneficial for people suffering from problems caused by procrastination. Second, the degree of therapist contact will have a positive effect on treatment outcome as procrastination can be partially explained as a self-regulatory failure. Third, an Internet based CBT intervention is presumed to be an effective way to administer treatment for procrastination, which is considered highly important, as the availability of adequate care is limited. The current study is therefore believed to render significant knowledge on the treatment of procrastination, as well as providing support for the use of Internet based CBT for difficulties due to delayed tasks and commitments.

**Conclusions:**

To our knowledge, the current study is the first clinical trial to examine the effects of CBT for procrastination, and is assumed to render significant knowledge on the treatment of procrastination, as well as investigating whether it can be delivered via the Internet.

**Trial Registration:**

ClinicalTrials.gov: NCT01842945; http://clinicaltrials.gov/show/NCT01842945 (Archived by WebCite at http://www.webcitation.org/6KSmaXewC).

## Introduction

### Defining Procrastination

Procrastination is defined as “to voluntarily delay an intended course of action despite expecting to be worse-off for the delay” [1]. It involves the postponement of initiating or completing a task or commitment until the last minute, after a predetermined deadline, or indefinitely. Albeit similar to difficulties prioritizing or being self-assertive, procrastination requires an active choice between competing activities, in which one is being avoided in favor of the other [2]. A common explanation for procrastination is based on learning theory and research on motivation and goal setting [3]. According to this perspective, procrastination is the result of an interaction between four variables: (1) expectancy, (2) value, (3) impulsiveness, and (4) time, also known as the procrastination equation [1]. Procrastination is a common behavioral pattern among adolescents and adults, and is presumed to be on the rise because of growing demands on individual responsibility and work flexibility, as well as greater availability of modern information technology [1]. Particularly vulnerable are young people studying at college or university, approximately half of this population suffers from difficulties due to procrastination, compared to 15%-20% in the general public [4]. Procrastination does not only cause problems concerning the task at hand, but has also been related with lower performance in school as well as work, decreased well-being, financial concerns, greater physical and mental illness, and fewer mental health seeking behaviors [5-8]. Both stress and anxiety are common among individuals who procrastinate recurrently, and is correlated with the degree of their difficulties [9,10].

### Procrastination Treatment

Even though procrastination is affiliated with great unrest among those afflicted, research on effective treatment methods is currently lacking. Cognitive behavior therapy (CBT) is often considered the treatment of choice, but since no clinical trials in the field of procrastination have been undertaken to date, its effectiveness is unknown [11]. The assumption that CBT might be beneficial for people suffering from difficulties due to procrastination is therefore primarily based on face validity and singe case studies. However, several of the cognitive and behavioral techniques used with people suffering from procrastination stem from treatment methods that have been proven effective for other psychiatric disorders. Behavioral activation is, for instance, originally developed for depression [12], but is often utilized in situations where a high degree of avoidance is causing great distress and decreased well being [13]. In relation to procrastination, behavioral activation helps the individual change an ongoing behavioral pattern so that tasks and commitments are approached rather than avoided. This usually requires some form of graded exposure considering the fact that procrastination is often reinforced by the unwillingness to experience discomfort [2]. Behavioral experiments can also be used to facilitate a reevaluation of work methods and presumptions regarding one’s own ability to achieve certain goals [14], aspects that are often characterized by either exaggerated optimism or pessimism among people who procrastinate [3]. This is often accompanied by other cognitive therapy techniques that aim to modify rigid and dysfunctional thoughts and assumptions that hinder the individual to behave more flexibly [15]. In addition, recent research findings in cognitive neuroscience and industrial psychology stress the importance of creating an effective work environment (eg, inhibiting the use of multitasking, decreasing the number of distractions, and preventing ego-depletion) [16-18]. In CBT this is generally referred to as stimulus control, and is regarded as beneficial for people who are afflicted by certain stimuli in their environment (eg, managing triggers for anxiety or avoiding situations that can cause problem behaviors such as substance abuse or deliberate self-harm) [19].

The effectiveness of specific CBT techniques for procrastination is however uncertain, thus making it unclear what mediates treatment outcome [11]. Since no clinical trials have yet to be carried out, it is not certain whether cognitive therapy, behavioral therapy, or a combination of both, is best suited for the treatment of procrastination. However, a recent meta-analysis indicates that several interventions related to addressing values and rewards, achieving stimulus control, enhancing goal-setting skills, and the utilization of success-spirals, could be beneficial for people suffering from procrastination [3]. Hence, certain CBT techniques, as well as methods from other disciplines, are assumed to be effective in treating procrastination.

### Trial Objectives and Purpose

The aim of this study is to examine the effects of CBT for procrastination, and to investigate whether it can be delivered via the Internet. The study will be based on a self-help book written by one of the authors (AR) [20], which for the purpose of the current research project has been divided into 10 modules that are to be delivered weekly to the participants. There will be two treatment groups used, one with therapist contact and one without, as well as a wait-list control group. It is assumed that the treatment group with therapist contact will be superior to the treatment group receiving no therapist contact since procrastination can be partially explained as a self-regulatory failure. Both treatment groups are presumed to be superior to the wait-list control.

## Methods

### Participants in the Sample

The current study is a randomized controlled trial (NCT01842945) with a total sample size of 150 participants divided into three groups; treatment with therapist contact (50 participants), treatment without therapist contact (50 participants), and wait-list control (50 participants). For ethical reasons the participants in the control group will gain access to the same intervention following the 10-week treatment period, albeit without therapist contact.

### The Intervention

There will be 10 modules from a self-help book on procrastination administered weekly to the participants via the Internet. These will contain psychoeducation, including information on the mechanisms underlying procrastination, different aspects affecting motivation, the concept of ego-depletion and mental fatigue, as well as a basic rationale of CBT. The modules will also include several techniques commonly used in the treatment of procrastination (eg, behavioral activation, graded exposure, behavioral experiments, identifying and testing rigid beliefs and assumptions, and stimulus control) [3,11,15]. The final module will focus on relapse prevention in order to successfully maintain behavior change [21].

The participants are instructed to study the material and carry out the assignments in each module. This consists of writing down any thoughts and ideas that arise from reading the provided psychoeducation, as well as carrying out different exercises each week (eg, goal-setting, time scheduling, identifying distractions, value clarification, and analyzing behavior patterns. For those participants receiving therapist contact, the completed assignments are retrieved at the end of every week in order to get continuous feedback on their work. This condition is assumed to be superior to those participants that are given each module without therapist contact as procrastination is maintained by difficulties concerning time management and task avoidance [3]. In other words, the presence of an external source of control (ie, a therapist contact) is expected to result in greater treatment outcome, as it will ensure that the participants undertake each module and practice relevant techniques. Similar to homework assignments in traditional face-to-face CBT, which has been found to increase both learning and adherence [22], receiving therapist contact is assumed to improve compliance throughout the treatment period. The feedback from a therapist contact can also be considered a source of positive reinforcement that many participants might lack when working on their day-to-day tasks and commitments [19]. However, even without a therapist contact participants are assumed to gain significant improvement as prior studies of Internet based CBT indicate that also a minimal level of therapist contact can have a positive effect on treatment outcome [23,24].

Internet based CBT has previously been found to be an effective way to administer treatment for several psychiatric conditions [25]. This includes depression [26], social phobia [27], panic disorder [28], generalized anxiety disorder [29], insomnia [30], post-traumatic stress disorder [31], tinnitus [32], and pathological gambling [33] among others. Internet based CBT is considered having several advantages over traditional face-to-face treatment (eg, higher cost-effectiveness, increased accessibility, greater possibility to reach patients in remote locations, and lower attrition rates) [34].

### Control Condition

The participants in the control group will be on a wait-list throughout the 10-week treatment period, and this group is expected to be inferior to the other two treatment groups. For ethical reasons the participants in the control group will gain access to the same intervention following the 10-week treatment period, albeit without therapist contact.

### Sample Size

The current study includes 150 participants randomized into three groups (ie, 50 participants in each group). Since no clinical trials in the field of procrastination have been undertaken, there are no previous effect sizes to take into consideration. However, 50 participants in each group are deemed sufficient to find clinically significant differences.

### Referral and Recruitment

Participants will be recruited through advertisements in newspapers, other media, and on the Internet. Only people residing in Sweden, having Internet access, and suffering from difficulties due to procrastination will be included in the study.

### Inclusion Criteria

Participants will be included in the study if their primary difficulties are caused by chronic and severe procrastination. However, since procrastination is not considered a psychiatric condition there will be no need to utilize diagnostic criteria (ie, Diagnostic and Statistical Manual of Mental Disorders Fourth edition, DSM-IV) [35], or a structured clinical interview (ie, Structured Clinical Interview for DSM-IV) [36]. In order to determine and evaluate the severity of procrastination several primary outcome measures will be used (eg, the Pure Procrastination Scale, PPS; the Irrational Procrastination Scale, IPS; and the Susceptibility to Temptation Scale; STS) [37]. To date, none of the primary outcome measures have clinically established cut-offs. However, greater than 32 points on the IPS have been found to distinguish more severe cases of procrastination (ie, top 25%) [1], and will therefore be used as cut-off for inclusion.

### Exclusion Criteria

In order to be included in the study participants are required to have a Swedish residency, to be at least 18 years of age, and fluent in Swedish, as well as having a computer with Internet access. Participants are not allowed to have an ongoing psychotherapy, and in case of taking psychotropic medication, the dose must have been stabilized for at least three months prior to entering treatment. Psychiatric conditions are not criteria for exclusion as long as procrastination is the primary problem. However, participants with severe depression (> 30 points on the Montgomery Åsberg Depression Rating Scale Self-report version, MADRS-S) [38], suicidal ideation, neuropsychiatric conditions (ie, Attention deficit hyperactivity disorder and Attention deficit disorder) misuse of alcohol or drugs, bipolar disorder, schizophrenia, and psychosis will be excluded from the study.

### Informed Consent

Participation will require a written informed consent.

### Withdrawal

Participation can be withdrawn at any time during the treatment period without specifying a reason behind the decision. In addition, should the condition of a participant deteriorate, supervising clinicians may choose to end the participation prematurely and direct the participant to other health care services.

### Safety Monitoring and Reporting

The current study adheres to the Swedish Personal Data Act [39]. The data being stored will be encrypted, and all participants will receive an auto-generated identification code in order to log on to a secure online interface (eg, 1234abcd), thus ensuring the anonymity of the participants during the analysis of the results. All communication with the participants will take place within the secure online interface that requires an electronic identification (ie, Secure Sockets Layer Certificates). Only reminders to log on to the secure online interface will be sent to the participants’ private email. At the postassessment, and at all subsequent assessment points, questions probing for adverse events will be used [40].

### Primary Outcomes

Primary outcome measures will be self-assessment of procrastination using a Swedish version of the PPS [37], the IPS [37], and the STS [37]. The PPS features 12 items measuring the prevalence of procrastination, and was originally developed to improve the validity of several different procrastination scales [37]. The items used in the PPS have a reliability of .92, and the PPS shows improved convergent validity with other related measures. The IPS features nine items measuring the degree of irrational delay causing procrastination. The items used in the IPS have a reliability of .91, and the IPS correlates together with the PPS at .96, allowing them to be used as parallel forms and share validation efforts [37]. The STS features 11 items measuring the susceptibility to temptation, which can affect the ability to follow through a task or commitment. The items in the STS have a reliability of .89, and demonstrate correlation with both the PPS and the IPS. However, since none of the primary outcome measures contain established cut-offs, clinical assessment will be used to determine treatment outcome. Participants will complete the primary outcome measures before commencing the treatment period, immediately upon completion of the treatment, and at the 12-month follow-up. Participants, including those on wait-list control, will also complete the IPS each week during the treatment period, allowing continuous measurement and increasing the statistical power.

### Secondary Outcomes

Secondary outcome measures will be self-assessment of depression, anxiety, and quality of life using the MADRS-S [41], Generalized Anxiety Disorder Assessment 7-item (GAD-7) [42], and Quality of Life Inventory (QOLI) [43]. MADRS-S is a self-assessment version of MADRS, and features nine items measuring changes in mood, anxiety, sleeping patterns, appetite, concentration, initiative, emotional engagement, pessimism, and attitude towards life [38]. MADRS is designed to be particularly sensitive to treatment effects, and shows high correlations, from *r*=.80 to .94, between expert ratings and self-reports [44]. GAD-7 features seven items for assessing anxiety, and screening for generalized anxiety disorder. GAD-7 has yielded good internal consistency, Cronbach alpha .92, and a good factorial structure, 69% to 81% of variance explained [42]. QOLI features 32 items concerning 16 areas of life rated by the subject concerning importance and satisfaction. QOLI has yielded good internal consistency, Cronbach alpha between .77 and .89, as well as one month test-retest reliability, from *r*=.80 to .91 [43,45]. Participants will complete the secondary outcome measures before commencing the treatment period, immediately upon completion of the treatment, and at the 12-month follow-up.

### Analysis

The study design will allow multiple comparisons-the two treatment groups, with or without therapist contact, will be compared to the wait-list control (separately and together) at posttreatment. In all subsequent analyses, the treatment groups will include those from the control group later randomized to interventions, which will increase the statistical power. The two treatment groups will be contrasted with the control group. Further, the treatment group with therapist contact (group 1) will be contrasted with the group without therapist contact (group 2). At the 12-month follow-up, within-group comparisons will be made with previous results. A number of statistical analyses will be deployed, all of which will be analyzed by the intention-to-treat approach. Treatment outcomes will be examined using mixed-effect models, Bonferroni-correcting for multiple comparisons. This method is deemed preferable to uni- and multivariate repeated measures of variance [46]. Standard missing data analysis (eg, *t* tests, chi-square of severity and sex) will be utilized to determine if unexpected missing data due to participant drop out are random or not.

All outcome measures will be completed directly by the participants via an online interface, minimizing the risk of data loss or data distortion [47,48]. Data will be stored encrypted and in unidentifiable form using an auto-generated identification code.

### Ethics

The regional Ethical Board (Dnr 2013/974-3175) has approved the current study. In the application, the following potential ethical issues were addressed. First, great consideration will be taken not to include participants suffering from any other primary diagnosis such as severe depression, suicidal ideation, or other disorders in immediate need of treatment. Should the condition of a participant deteriorate significantly during the treatment period, supervising clinicians may choose to end the participation prematurely and direct the participant to other health care services. Second, no adverse side effects of CBT interventions are assumed to occur. However, this will nonetheless be investigated by asking standard treatment side effects questions at the posttreatment measurements [49]. Third, when using the Internet to communicate and administer interventions, the privacy of the participants is of the greatest importance [50]. As mentioned earlier, participants will therefore use anonymous auto-generated identification codes to interact with a secure online interface, and only reminders will be sent to the participants’ private email.

## Results

### The Current Study

The current study is a randomized controlled trial examining the effects of Internet based CBT for procrastination and is believed to result in several important findings. First, research on procrastination has mainly focused on underlying mechanisms affecting motivation, often involving the study of personality constructs and different demographic variables [3,11]. Although some factors have been found to mediate difficulties due to procrastination (eg, neuroticism, self-efficacy, self-esteem, depression, age, and gender) [15,51-53,54,55], the results are far from consistent and the implications not always clear [3]. Meanwhile, research on the treatment of procrastination is scarce, affecting the possibility of receiving adequate care. CBT is often considered the treatment of choice, even though no clinical trials have yet been carried out [11]. The aim of this study will be to test the effects of CBT for procrastination, thereby generating significant knowledge on what interventions can be used in the treatment of difficulties due to unattended tasks and commitments.

Second, previous research on Internet based CBT has found it to be an effective way to administer treatment for several psychiatric conditions. It is therefore presumed to be beneficial for people suffering from procrastination. The aim of this study will be to investigate whether CBT for procrastination can have a positive effect on treatment outcome if delivered via the Internet, and to test whether or not receiving weekly therapist contact can influence treatment outcome. This is considered highly important as the availability of adequate care is limited, and should it prove effective in alleviating difficulties due to procrastination, it would greatly increase the possibility of receiving treatment regardless of geographic location.

### Trial Status

The recruitment of participants to the current study was completed in August 2013. The treatment period commenced in September for both treatment groups and ends in December the same year. The wait-list control will begin treatment two weeks later and finish in February 2014. Preliminary results will be available later the same month. Follow-up will be carried through 12 months later (ie, December 2014 for the two treatment groups and February 2015 for the control group).

## Discussion

### Summary

To our knowledge, the current study is the first clinical trial to examine the effects of CBT for procrastination, and is assumed to render significant knowledge on the treatment of procrastination. The study also investigates whether Internet based CBT, either with or without support from a therapist contact, can be beneficial for people suffering from difficulties due to procrastination.

### Study Limitations

The current study has some limitations that need to be recognized. First, due to the fact that procrastination is not considered a psychiatric condition there may arise difficulties concerning the evaluation of clinically significant change. In order to assess the effects of the interventions being employed, self-assessments are utilized to determine the severity of procrastination among the participants. However, since none of the primary outcome measures contain clinically established cut-offs, the Reliable Change Index will be used to determine clinical significant change [56]. This might affect the validity and reliability of the results, but is compensated for by the use of secondary outcome measures (eg, MADRS-S, GAD-7 and QOLI), as procrastination often causes great unrest and decreased well being among those afflicted. Second, the use of a Swedish version of the primary outcome measures can also influence the validity of the results, which is why an authorized translator is hired to ensure that no significant loss of meaning is being made during the translation process. Third, another limitation in need of recognition is the involvement of master students in clinical psychology as online therapists. To make up for the students’ lack of training and experience, an experienced licensed clinical psychologist and psychotherapist will supervise all involvement in the study.

### Conclusions

Procrastination is a common psychological problem causing major distress, especially among the student population. CBT is deemed the treatment of choice and is evaluated in a clinical trial conducted entirely over the Internet.

## Abbreviations

CBT: cognitive behavior therapy
DSM-IV: The Diagnostic and Statistical Manual of Mental Disorders Fourth edition
GAD-7: Generalized Anxiety Disorder Assessment 7-item
IPS: Irrational Procrastination Scale
MADRS: Montgomery Åsberg Depression Rating Scale
MADRS-S: Montgomery Åsberg Depression Rating Scale Self-report version
PPS: Pure Procrastination Scale
QOLI: Quality of Life Inventory
STS: Susceptibility to Temptation Scale

## References

[1] Steel P (2011). How to stop putting things off and start getting stuff done. The Procrastination Equation.

[2] Dryden W (2000). Overcoming procrastination.

[3] Steel P (2007). The nature of procrastination: a meta-analytic and theoretical review of quintessential self-regulatory failure. Psychol Bull.

[4] Burka JB, Yuen LM (2009). Procrastination: Why You Do It, What to Do About It Now.

[5] Steel P, Brothen T, Wambach C (2001). Procrastination and personality, performance, and mood. Personality and Individual Differences.

[6] Sirois FM, Melia-Gordon ML, Pychyl TA (2003). "I'll look after my health, later": an investigation of procrastination and health. Personality and Individual Differences.

[7] Bogg T, Roberts BW (2004). Conscientiousness and health-related behaviors: a meta-analysis of the leading behavioral contributors to mortality. Psychol Bull.

[8] Stead R, Shanahan MJ, Neufeld RW (2010). “I’ll go to therapy, eventually”: Procrastination, stress and mental health. Personality and Individual Differences.

[9] Rothblum ED, Solomon LJ, Murakami J (1986). J Couns Psychol.

[10] Tice DM, Baumeister RF (1997). Longitudinal study of procrastination, performance, stress, and health: the costs and benefits of dawdling. Psychological Science.

[11] Pychyl TA, Flett GL (2012). Procrastination and self-regulatory failure: an introduction to the special issue. J Rat-Emo Cognitive-Behav Ther.

[12] Ferster CB (1973). A functional analysis of depression. Am Psychol.

[13] Mazzucchelli TG, Kane RT, Rees CS (2010). Behavioral activation interventions for well-being: A meta-analysis. J Posit Psychol.

[14] Bennett-Levy J (2004). Oxford guide to behavioural experiments in cognitive therapy.

[15] Flett GL, Stainton M, Hewitt PL, Sherry SB, Lay C (2012). Procrastination automatic thoughts as a personality construct: an analysis of the procrastinatory cognitions inventory. J Rat-Emo Cognitive-Behav Ther.

[16] Baumeister RF, Gailliot M, DeWall CN, Oaten M (2006). Self-regulation and personality: how interventions increase regulatory success, and how depletion moderates the effects of traits on behavior. J Pers.

[17] Brennan A, Chugh JS, Kline T (2002). Traditional versus Open Office design: a longitudinal field study. Environment and Behavior.

[18] Lorist MM, Klein M, Nieuwenhuis S, De Jong R, Mulder G, Meijman TF (2000). Mental fatigue and task control: planning and preparation. Psychophysiology.

[19] Cooper JO, Heron TE, Heward WL (2007). Applied behavior analysis.

[20] Rozental A, Wennersten L (2014). Dansa på deadline.

[21] Donovan DM, Marlatt GA (2007). Relapse Prevention, Second Edition: Maintenance Strategies in the Treatment of Addictive Behaviors.

[22] Kazantzis N, Deane FP, Ronan KR (2000). Homework assignments in cognitive and behavioral therapy: A meta-analysis. Clin Psychol-Sci Pr.

[23] Carlbring P, Nilsson-Ihrfelt E, Waara J, Kollenstam C, Buhrman M, Kaldo V, Söderberg M, Ekselius L, Andersson G (2005). Treatment of panic disorder: live therapy vs. self-help via the Internet. Behav Res Ther.

[24] Nordin S, Carlbring P, Cuijpers P, Andersson G (2010). Expanding the limits of bibliotherapy for panic disorder: randomized trial of self-help without support but with a clear deadline. Behav Ther.

[25] Cuijpers P, Donker T, van Straten A, Li J, Andersson G (2010). Is guided self-help as effective as face-to-face psychotherapy for depression and anxiety disorders? A systematic review and meta-analysis of comparative outcome studies. Psychol Med.

[26] Andersson G, Bergström J, Holländare F, Carlbring P, Kaldo V, Ekselius L (2005). Internet-based self-help for depression: randomised controlled trial. Br J Psychiatry.

[27] Carlbring P, Gunnarsdóttir M, Hedensjö L, Andersson G, Ekselius L, Furmark T (2007). Treatment of social phobia: randomised trial of Internet-delivered cognitive-behavioural therapy with telephone support. Br J Psychiatry.

[28] Carlbring P, Ekselius L, Andersson G (2003). Treatment of panic disorder via the Internet: a randomized trial of CBT vs. applied relaxation. J Behav Ther Exp Psychiatry.

[29] Paxling B, Almlöv J, Dahlin M, Carlbring P, Breitholtz E, Eriksson T, Andersson G (2011). Guided Internet-delivered cognitive behavior therapy for generalized anxiety disorder: a randomized controlled trial. Cogn Behav Ther.

[30] Espie CA, Kyle SD, Williams C, Ong JC, Douglas NJ, Hames P, Brown JS (2012). A randomized, placebo-controlled trial of online cognitive behavioral therapy for chronic insomnia disorder delivered via an automated media-rich Web application. Sleep.

[31] Spence J, Titov N, Dear BF, Johnston L, Solley K, Lorian C, Wootton B, Zou J, Schwenke G (2011). Randomized controlled trial of Internet-delivered cognitive behavioral therapy for posttraumatic stress disorder. Depress Anxiety.

[32] Hesser H, Gustafsson T, Lundén C, Henrikson O, Fattahi K, Johnsson E, Zetterqvist Westin V, Carlbring P, Mäki-Torkko E, Kaldo V, Andersson G (2012). A randomized controlled trial of Internet-delivered cognitive behavior therapy and acceptance and commitment therapy in the treatment of tinnitus. J Consult Clin Psychol.

[33] Carlbring P, Smit F (2008). Randomized trial of Internet-delivered self-help with telephone support for pathological gamblers. J Consult Clin Psychol.

[34] Carlbring P, Andersson G (2006). Internet and psychological treatment. How well can they be combined?. Computers in Human Behavior.

[35] American Psychiatric Association (2000). Diagnostic and statistical manual of mental disorders: DSM-IV.

[36] Sheehan DV, Lecrubier Y, Sheehan KH, Amorim P, Janavs J, Weiller E, Hergueta T, Baker R, Dunbar GC (1998). The Mini-International Neuropsychiatric Interview (M.I.N.I.): the development and validation of a structured diagnostic psychiatric interview for DSM-IV and ICD-10. J Clin Psychiatry.

[37] Steel P (2010). Arousal, avoidant and decisional procrastinators: Do they exist?. Personality and Individual Differences.

[38] Svanborg P, Asberg M (2001). A comparison between the Beck Depression Inventory (BDI) and the self-rating version of the Montgomery Asberg Depression Rating Scale (MADRS). J Affect Disord.

[39] Datainspektionen (1998). Personal Data Act (1998:204).

[40] Parker G, Fletcher K, Berk M, Paterson A (2013). Development of a measure quantifying adverse psychotherapeutic ingredients: the Experiences of Therapy Questionnaire (ETQ). Psychiatry Res.

[41] Svanborg P, Asberg M (1994). A new self-rating scale for depression and anxiety states based on the Comprehensive Psychopathological Rating Scale. Acta Psychiatr Scand.

[42] Spitzer RL, Kroenke K, Williams JB, Löwe B (2006). A brief measure for assessing generalized anxiety disorder: the GAD-7. Arch Intern Med.

[43] Frisch MB, Cornell J, Villanueva M, Retzlaff PJ (1992). Clinical validation of the Quality of Life Inventory. A measure of life satisfaction for use in treatment planning and outcome assessment. Psychological Assessment.

[44] Montgomery SA, Asberg M (1979). A new depression scale designed to be sensitive to change. Br J Psychiatry.

[45] Lindner P, Andersson G, Ost LG, Carlbring P (2013). Validation of the Internet-Administered Quality of Life Inventory (QOLI) in different psychiatric conditions. Cogn Behav Ther.

[46] Gueorguieva R, Krystal JH (2004). Move over ANOVA: progress in analyzing repeated-measures data and its reflection in papers published in the Archives of General Psychiatry. Arch Gen Psychiatry.

[47] Carlbring P, Brunt S, Bohman S, Austin D, Richards J, Öst L, Andersson G (2007). Internet vs. paper and pencil administration of questionnaires commonly used in panic/agoraphobia research. Computers in Human Behavior.

[48] Thorndike FP, Carlbring P, Smyth FL, Magee JC, Gonder-Frederick L, Ost L, Ritterband LM (2009). Web-based measurement: Effect of completing single or multiple items per webpage. Computers in Human Behavior.

[49] Linden M (2013). How to define, find and classify side effects in psychotherapy: from unwanted events to adverse treatment reactions. Clin Psychol Psychother.

[50] Proudfoot J, Klein B, Barak A, Carlbring P, Cuijpers P, Lange A, Ritterband L, Andersson G (2011). Establishing guidelines for executing and reporting Internet intervention research. Cognitive Behaviour Therapy.

[51] Brown RT (1992). Journal of College Student Psychotherapy.

[52] Bandura A (1997). Self-efficacy: the exercise of control.

[53] Judge TA, Bono JE (2001). Relationship of core self-evaluations traits--self-esteem, generalized self-efficacy, locus of control, and emotional stability--with job satisfaction and job performance: a meta-analysis. J Appl Psychol.

[54] Baumeister RF, Heatherton TF, Tice DM (1994). Losing control: how and why people fail at self-regulation.

[55] Feingold A (1994). Gender differences in personality: a meta-analysis. Psychol Bull.

[56] Jacobson NS, Truax P (1991). Clinical significance: a statistical approach to defining meaningful change in psychotherapy research. J Consult Clin Psychol.

